# The regulatory network of the chemokine CCL5 in colorectal cancer

**DOI:** 10.1080/07853890.2023.2205168

**Published:** 2023-05-04

**Authors:** Xin-Feng Zhang, Xiao-Li Zhang, Ya-Jing Wang, Yuan Fang, Meng-Li Li, Xing-Yu Liu, Hua-You Luo, Yan Tian

**Affiliations:** a Department of Gastrointestinal and Hernia Surgery, First Affiliated Hospital of Kunming Medical University, Kunming, China; b Department of General Surgery, Third Medical Center of PLA General Hospital, Beijing, China; c Organ Transplant Department, First Affiliated Hospital of Kunming Medical University, Kunming, China; d Honghui Hospital affiliated to Yunnan University, Kunming, China

**Keywords:** CCL5, colorectal cancer, immune regulation, immunotherapy, tumor microenvironment

## Abstract

The chemokine CCL5 plays a potential role in the occurrence and development of colorectal cancer (CRC). Previous studies have shown that CCL5 directly acts on tumor cells to change tumor metastatic rates. In addition, CCL5 recruits immune cells and immunosuppressive cells into the tumor microenvironment (TME) and reshapes the TME to adapt to tumor growth or increase antitumor immune efficacy, depending on the type of secretory cells releasing CCL5, the cellular function of CCL5 recruitment, and the underlying mechanisms. However, at present, research on the role played by CCL5 in the occurrence and development of CRC is still limited, and whether CCL5 promotes the occurrence and development of CRC and its role remain controversial. This paper discusses the cells recruited by CCL5 in patients with CRC and the specific mechanism of this recruitment, as well as recent clinical studies of CCL5 in patients with CRC.

**Key Messages**
1. CCL5 plays dual roles in colorectal cancer progression.
2. CCL5 remodels the tumor microenvironment to adapt to colorectal cancer tumor growth by recruiting immunosuppressive cells or by direct action.
3. CCL5 inhibits colorectal cancer tumor growth by recruiting immune cells or by direct action.

## 1. Introduction

Colorectal cancer (CRC) is the third most common solid tumor in the world and one of the leading causes of cancer-related death [1]. The development of immunotherapy, including immune checkpoint inhibitors, chimeric antigen receptor T cells and tumor vaccines, has led to great progress in cancer treatment by unleashing the beneficial killing ability of T-cells in a variety of cancers [2]. CRC has shown considerable resistance to multiple immunotherapies, especially blocking immune checkpoints, which have exhibited therapeutic effects on many other types of cancer [3]. Cytotoxic CD8^+^ T cells constitute a main group of effector immune cells in antitumor immunity. The amount of intratumoral infiltration of CD8^+^ T cells is regarded as a positive prognostic indicator for many human cancer types [4]. For example, cancers with a high tumor mutation burden and genomic instability, such as microsatellite unstable CRC, high CD8^+^ T-cell infiltration has been significantly associated with a good prognosis [5]. Although there are a large number of circulating active CD8^+^ T cells in tumor patients, the lack of CD8^+^ T cells in the central tumor area has become a major obstacle to immunotherapy for solid tumors, especially in patients with CRC [6]. The enhancement of CD8^+^ T cell-mediated antitumor immunity and the transport of CD8^+^ T cells to the tumor sites are essential for effective cancer treatment, abd chemokines regulate T-cell aggregation in solid tumors [7].

Increasing evidence shows that chemokines play important roles in tumor growth and metastasis. Different members of the chemokine family promote or inhibit tumor growth by promoting or inhibiting tumor angiogenesis [8]. With molecular weights ranging from 7 to 15 kDa, chemokines are the main proteins secreted to the extracellular space. To date, 50 chemokines have been identified [9]. Although their main role is recruitment and activation of the immune response, their important role in the process of tumor cell invasion, metastasis and immune response escape has been increasingly recognized [10]. Most tumors promote their own growth by recruiting stromal cells to shape the local chemokine network [11]. Among the known human chemokines, CCL4, CCL5, CXCL9 and CXCL10 are closely related to CD8^+^ T-cell infiltration [12]. Among these proteins, CCL5 affects tumor progression in an autocrine or a paracrine manner, such as by directly affecting cancer cell proliferation, migration and survival through its autocrine function or by indirectly recruiting inflammatory cells into the tumor microenvironment (TME)through paracrine function, thus shaping the TME for its own survival [13]. In this study, we explored the role played by the chemokine CCL5in the occurrence and development of CRC. Herein, we explain the relevant regulatory mechanism of CCL5, to provide a reference for clinical research.

## 2. CCL5 and its receptors

**Figure 1.**
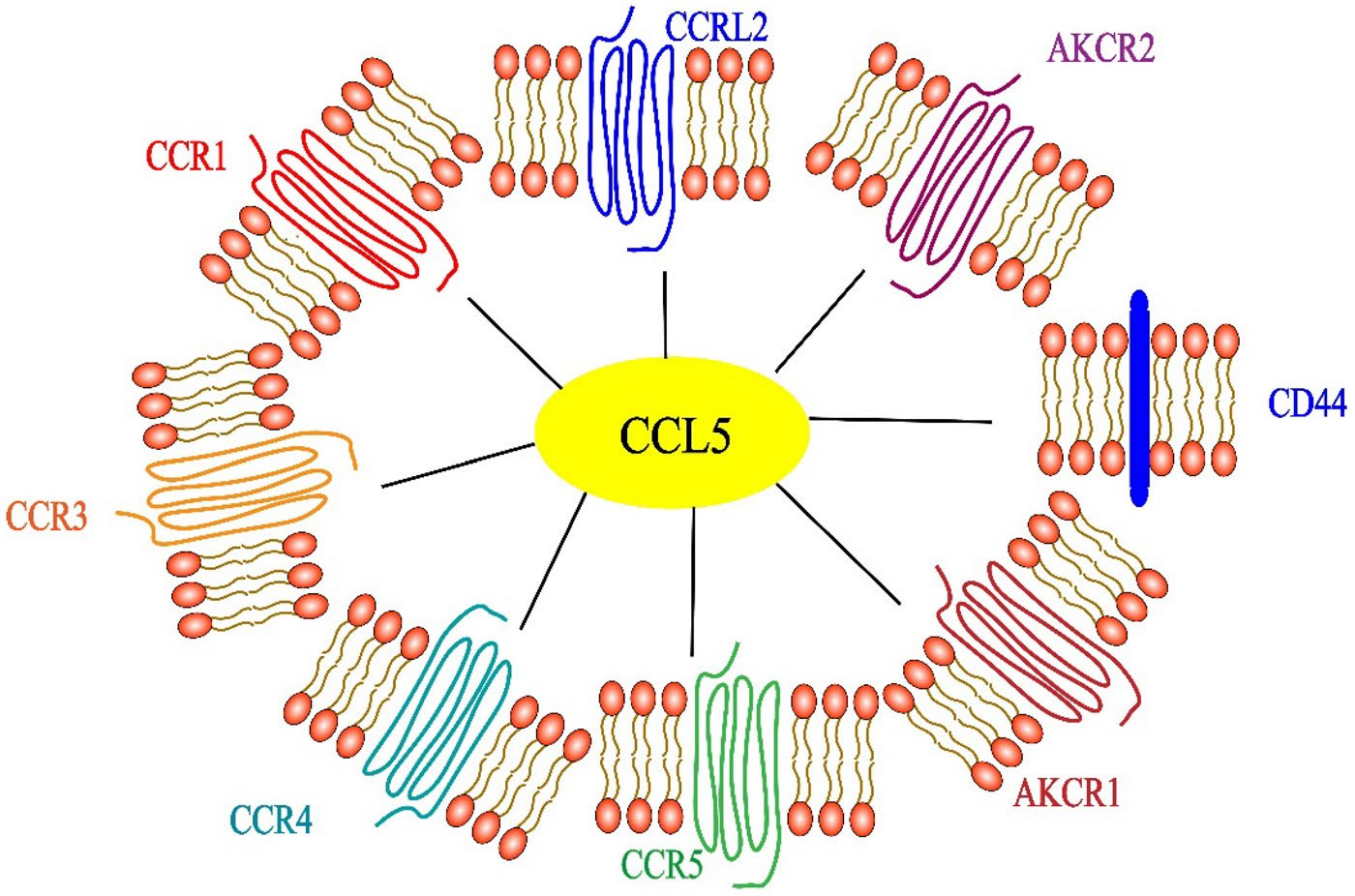
Receptors related of the chemokine CCL5. In addition to the cognate receptor CCR5, specific receptors include CCR1, CCR3 and CCR4. Noncanonical receptors include ACKR1, ACKR2, CCRL2 and CD44.

The chemokine (cysteine-cysteine motif) ligand 5 (CCL5/RANTES) is a secretory small molecule protein that is expressed in the blood and TME. It was discovered in 1988 by Schall TJ et al. [14] using a cDNA library enriched with T-cell-specific sequences [14]. The gene product of CCL5 is 10 kDa (91 amino acids), and the signal peptide is approximately 8 kDa after cleavage. Of the 68 residues, 4 are cysteine residues and harbor no N-linked glycosylation sites [14]. CCL5 is a member of the CC chemokine family because it has two pairs of adjacent cysteine residues near the amino terminus and is expressed and secreted by macrophages, T cells, tubular epithelial cells, synovial fibroblasts and certain types of cancer cells [15]. As a chemokine, it mediates the involvement of a variety of cells in the inflammatory response, including the transport and homing of T cells, monocytes and NK cells [16,17]. The regulation of CCL5 expression is complex. IFN, IL-1 and TNF-α induce the activation of CCL5 through transcription factors, including NF-kB, interferon regulatory factor 1, interferon regulatory factor 3, interferon regulatory factor 7, STAT1 or STAT3 [18–20] (Figure 1).

CCL5 induces a cell-type specific signaling response by binding to specific G protein-coupled receptors CCR1, CCR3, CCR4 and CCR5 on the surface of target cells [21,22]. CD44 is the coreceptor of CCL5 [23]. The atypical receptor (nonsignal receptors such as ACKR1, ACKR2 and CCRL2) also interact with CCL5 [24]. Among these receptors, CCR1 and CCR3 reside exclusively on the surface of immune cells [25], but CCR5 expression has been found in all CRC samples [26]. CCR5 is the main signal receptor of CCL5 and a potential drug target for most immune diseases [27]. CCL5 has been proven to be the main coreceptor of human immunodeficiency virus-1 (HIV-1), which is very important for the pathogenesis of HIV-1 [28]. There have been a large number of studies devoted to the role of CCR5 in HIV-1 infection. Specifically, HIV-1 binds to CD4^+^ T cells and enters CD4^+^ T cells with CD4 being the main receptor, while CCR5 is also a necessary receptor. The small-molecule inhibitor maraviroc and humanized monoclonal antibody leronlimab are CCR5 antagonists that inhibit the entry of the HIV-1 virus into CD4^+^ T cells [29]. At present, the most promising method to block CCR5 is by administering the drug maraviroc, which is an allosteric inverse agonist of CCR5 [30,31]. However, recent studies have been more focused on cancer than on HIV-1 treatment. CCR5 is overexpressed in many types of cancers. Experiments *in vivo* and *in vitro* have shown that acquired resistance to trastuzumab in cancer patients was inhibited by maraviroc blocking CCL5/CCR5 binding [31]. Maraviroc inhibits the binding of CCL5 and CCR5 by reducing the proliferation and metastasis of cancer cells [32]. Although its potential role in cancer progression has been reported in some studies of various tumor types, including CRC, the CCL5/CCR5 axis has not been extensively studied in cancer treatment, and the role of CCL5 in CRC is still controversial. We believe that the main reason for the lack of clarity is because CCL5 is expressed in different cells and its specific receptors include CCR1, CCR3, CCR4 and CCR5 (see Table 1 for details of its receptor-related functions). Different cells can be recruited to participate in the inflammatory response; therefore, the effect of CCL5 on the immune environment is different in physiological and pathological processes, even in different cancer models and tissue types, as explained in detail in Table 2.

**Table 1.** Function of the CCL5 receptor.

Receptor	Function
CCR1	Highly expressed in human monocyte/macrophage [33] contribute to transendothelial migration of monocytes and T cells in atherogenic lesions and then promote disease progression [34] monocyte recruitment, not involved in apoptosis [35]
CCR3	Highly expressed in human eosinophils, basophils and mast cells [36,37] correlate with allergic diseases such as asthma, atopic dermatitis, and allergic rhinitis. And as a possible pathogenic mechanism of such diseases [38,39] tumor progression [40]
CCR4	Highly expressed in human T lymphocyte cells [41] play a central role in T cell migration to the thymus, and T cell maturation and education [42] correlate with leukemia and lymphoma, contributing to tumor survival [43] correlate with autoimmune disease such as multiple sclerosis, Primary Sjögren’s syndrome, asthma and atopic dermatitis and then promote disease progression [17,39,44, 45]
CCR5	Contribute to transendothelial migration of monocytes and T cells in atherogenic lesions [34] cancer progression: ①DNA damage repair [46] ②extracellular matrix remodelling [47,48] ③tumour progression and metastasis [49] ④cancer stem cell proliferation [50] ⑤autocrine/paracrine signalling [51] ⑥tumor angiogenesis [52] ⑦metabolic reprogramming [53] ⑧immunosuppression [54]

**Table 2.** Expression of CCL5 in human cells and its role.

Receptor	Cell type^a^	Mechanisms^b^	Disease	Ref.
	Microglia	Neuroinflammation (mTOR signaling)	Autism Spectrum Disorder	[55]
	Macrophages	Tumor progression and chemoresistance (inhibiting apoptosis)	Cholangiocarcinoma	[56]
	Macrophages	Immune escape (p65/STAT3-CSN5-PD-L1 pathway)	colorectal cancer	[57]
	Tumour tissues	Regulating the tumour immune microenvironment(CD8^+^ T cell infiltration)	colorectal neuroendocrine carcinoma	[58]
	Tumour tissues	Mast cell recruitment tumor progression	clear cell renal cell carcinoma	[59]
	Platelet	Thromboinflammatory responses(leukocyte chemoattraction and monocyte recruitment)	Essential Thrombocythemia	[60]
CCR1	Mesenchymal stem cells	Epithelial–mesenchymal transition and progression(CCL5/β-catenin/Slug pathway)	colorectal cancer	[61]
CCR1	White adipose tissue	Antiapoptotic (Erk/Akt pathways) macrophage recruitment	Obesity	[62]
CCR3	Stromal cells	Tumor progression	Breast Cancer	[40]
CCR1 CCR3	Platelets Endothelium	Leukocyte recruitment	Atherosclerosis	[63]
CCR1 CCR3	Ovarian cancer stem-like cells	Tumor metastatic(CCL5/CCR1-CCR3/NF-KB/MMP-9)	ovarian cancer	[64]
CCR4	CCR9^+^effector CD8^+^ T cells	Autoimmune damage	Primary Sjögren’s syndrome	[17,65]
CCR1 CCR5	Cancer cell	Recruits blood monocytes	Diffuse large B-cell lymphoma	[66]
CCR1 CCR5	Cancer cell	Development and metastasis of Breast Cancer(macrophage recruitment)	Breast Cancer	[67]
CCR3 CCR5	Brain microvascular endothelial cells	Zika virus pathogenesis and spread(CCL5/CCR3-CCR5/ERK1/2)	Microcephaly	[18]
CCR5	Colorectal cancer cell	Tumor progression (apoptosis of CD8 T cells, infiltration of Treg cells)	colorectal cancer	[50]
CCR5	Pancreatic cancer cells	Immunosuppression (recruit FOXP3^+^ Treg cells)	pancreatic ductal adenocarcinoma	[54,68]
CCR5	Glioblastoma Cells	Proliferation and invasion(Remodeling the tumor microenvironment)	Glioblastoma Cells	[48]
CCR5	Cancer cell	Acquired resistance to trastuzumab(ERK)	Breast Cancer	[31]
CCR5	Cutaneous neurofibromas cells	Nerve injury(macrophage recruitment YAP)	neurofibromatosis type 1(Cutaneous neurofibromas)	[69]
CCR5	Macrophages	Chemo-resistance and distant metastasis(STAT3-associated signaling)	prostatic cancer	[20,70]

For the first 6 rows, there are no receptors specified because the relevant receptors are not specified in these six literatures.

^a^Types of cells secreting CCL5.

^b^Specific mechanisms of CCL5 receptor cells and downstream activated pathways.

### 2.1. The relationship between immunosuppressive cells recruited by CCL5 and tumorigenesis and progression

**Figure 2.**
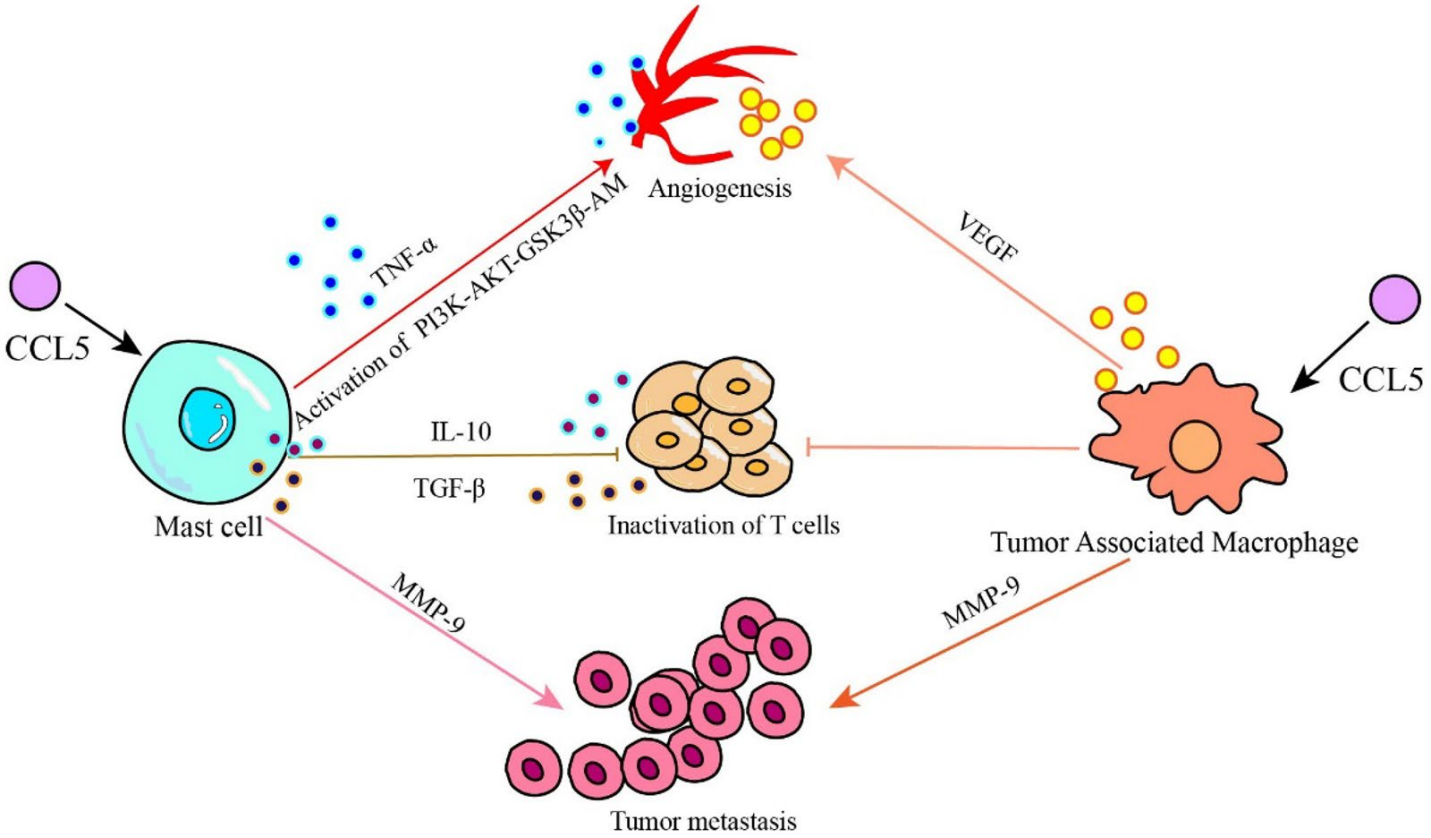
CCL5 promotes tumor progression by recruiting immunosuppressive cells. Mast cells recruited by CCL5 promote tumor neovascularization by secreting the cytokine TNF- α and activating the PI3K/AKT/GSK3 β/AM pathway; mast cells inhibit the activity of CD8+ T cells by secreting the cytokines IL-10 and TGF-β, and play immunosuppressive roles; mast cells and tumor-associated macrophages can directly promote tumor metastasis by secreting MMP-9. Tumor-associated macrophages are recruited by CCL5 to promote tumor neovascularization *via* the secretion VEGF; and TAMs directly inhibit the activity of CD8+ T cells.

CCL5 can recruit a variety of cell types to respond to inflammation, including monocytes, macrophages, mast cells, eosinophils, basophils and dendritic cells. It is also involved in the migration of leukocytes to inflammatory tissues and the proliferation and activation of natural killer (NK) cells [71,72]. These cells and cancer cells constitute the TME, and most immunosuppressive cells undergo metabolic reprogramming in the TME. The metabolic changes in these cells lead to immunosuppression of CD8^+^ T cells and limit the antitumor immune response in advanced cancer. Here, we discuss the role and efficacy of some immunosuppressive cells such as mast cells (MCs) and tumor-associated macrophages in tumorigenesis and development (Figure 2).

CCL5 is a chemoattractant for MCs. MCs are an immunosuppressive cells that inhibit the function of NK and other immune cells. MCs play a central role in the activation of gastrointestinal cells and endothelial cells, promoting tumor angiogenesis and progression. It has been reported that MCs play a role in promoting angiogenesis switching during tumor growth [73,74], and MCs and their secreted cytokines play important roles in inflammation-mediated tumorigenesis by regulating proinflammatory cytokines and inducible inflammatory enzymes [75]. The main mechanism involves MCs secretion of TNF- α, which is the factor that induces tumor angiogenesis, and MCs activate the PI3K/AKT/GSK3β/AM signaling pathway to promote tumor microangiogenesis [76]; MCs secrete excessive immunosuppressive cytokines such as IL-10 and TGF- β to inhibit the infiltration and function of CD8^+^ T cells, while CD8^+^ T cells are the key cell subsets that inhibit tumor growth and progression [59,77]; MCs also promote metastasis and escape of tumor cells from immune responses by upregulating matrix metalloproteinases (such as MMP9) [78]. Studies by Tanaka et al. [75] showed that mice lacking MCs were less susceptible to inflammation-related CRC [75].

**Figure 3.**
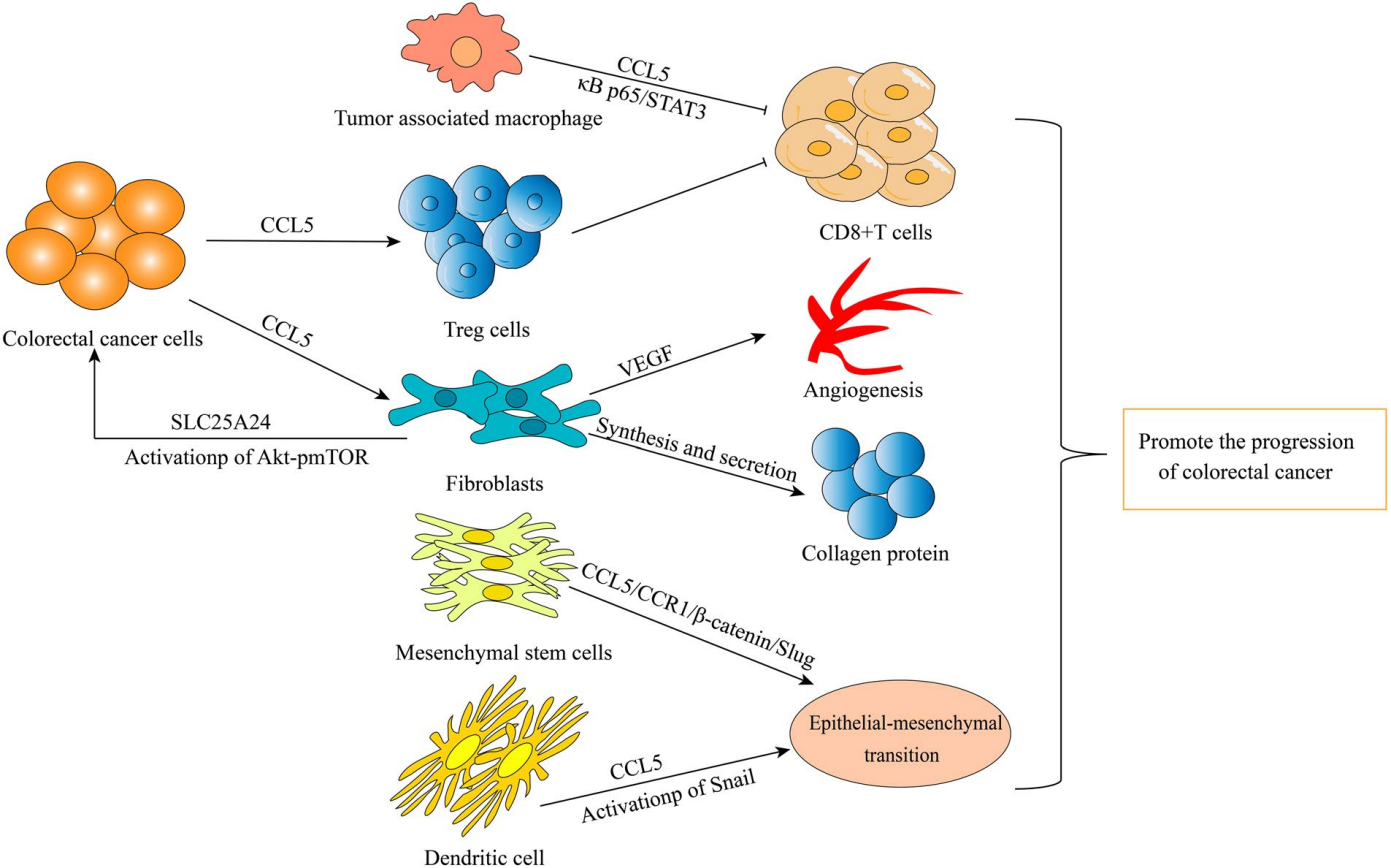
All kinds of cells in tumor microenvironment promote tumor progression in different ways: TAMs inhibit the activity of CD8+ T cells through CCL5/Kappa p65/STAT3 signaling pathway. Colorectal cancer cells recruit Treg cells to inhibit CD8^+^ T cell activity by secreting CCL5, or act on Flibroblasts by secreting CCL5. Flibroblasts promote tumor angiogenesis by secreting VEGF, synthesizing and secreting collagen protein, and activating SLC25A24 and Akt/pmTOR signaling pathways. Mesenchymal stem cells and dendritic cells promote stromal epithelial transformation by secreting CCL5.

CCL5 binds to the receptor CCR5 and plays a role similar to that of oncogenes that is, it promotes tumor growth, induces extracellular matrix remodeling, recruits immune cells and polarizes tumor-associated macrophages (TAMs) [64,79]. CCL5 directly induce TAMs to secrete MMP9 and promote tumor cell metastasis [80]. In the initial stage of cancer development, TAMs in the TME show typical activation or an M1-like phenotype and robust antitumor activity and secrete proinflammatory cytokines such as IL-1β, IL-6, IL-12 and IL-23 [81]. However, in the later stage of cancer development, when growth factors and anti-inflammatory cytokines such as IL-4, IL-10 and TGF-β are enriched in the TME, TAMs polarize to anti-inflammatory M2-like phenotype, which helps to maintain an immunosuppressive TME [82]. M2 TAMs constitute heterogeneous cell type that promotes malignant tumors through the production of angiogenic growth factors, extracellular matrix remodeling and immunosuppression [83]. The epigenetic modification of cancer cells promotes the transport of TAMs to the TME, resulting in high PD-1 protein expression on CD8^+^ T cells and tumor progression [84]. The expression of PD-1 on CD8^+^ T cells suppresses the activation of CD8^+^ T cells mediated by T-cell receptors [85]. Moreover, blocking PD-1/PD-L1 *in vivo* increases the phagocytosis by TAMs and reduces tumor growth, indicating that PD-1/PD-L1 therapy may exert a direct effect on TAMs [86]. The deletion of TAMs in the TME or inhibition of CSF1/CSF1R, the main survival ligand/receptor pair secreted by TAMs, significantly enhances the recruitment of CD8^+^ T cells and induces tumor regression [87] (Figure 3).

## 3. Regulation of protective antitumor immunity mediated by CCL5

Some of the results show that the expression of CCL5 in CRC tissue has an antitumor effect, and its mechanism is realized by recruiting immune cells to the TME. Among these cells, type-1 dendritic cells (cDC1s) and CD8^+^ T cells play important roles in antitumor immunity. Their abundance in tumor and their activation triggered by therapy may enhance antitumor immunity and increase the response of cancer patients to immunotherapy [88]. In fact, the increase in the rate of cDC1s infiltration into tumors and the protective effect it confers depend on the expression of CCL5 and CCR5. In addition, the expression of CCL5 and CCR5 is related to an increase in cDC1s in the TME and the prolonged overall survival time of cancer patients [89]. The specific mechanisms of cDC1s action in the TME include cDC1s absorption of dead tumor cells and the transport of tumor antigens to the lymph nodes that drain the tumor [90]. In addition to this transport, intratumoral cDC1s attract CD8^+^ T cells restimulating and attracting tumor-specific CD8^+^ T cells to play an antitumor immune role [91]. Other studies have shown that cDC1-derived IL-12 is important for the antitumor activity of NK cells [92]. In addition, infiltrating CD8^+^ T cells or other types of cells that produce CCL5 may maintain cDC1 recruitment [88]. This possibility suggests that the activation of CCL5/CCR5 signaling may enhance the antitumor immune effect of CRC cancer patients. CCL5 inhibits tumor growth by promoting the infiltration of antitumor immune cells cDC1s into the TME, producing a more effective antitumor immune response. In addittion, some studies have suggested that in CRC tissue, the increase in CCL5 level is positively correlated with the number and activity of CD8^+^ cytotoxic T cells. It has been suggested that the expression of CCL5 in CRC tissue has exerts an antitumor effect [93]. Therefore, some people think that cancer immunotherapy should be combined with a strategy to increas the expression of CCL5 in tumors, thereby enhancing the infiltration of various immune cells into the TME, to increase the therapeutic effect [94].

## 4. The role of CCL5 in the tumor microenvironment

Tumorigenesis is a multistage process that is usually initiated by activating oncogenes or suppressing mutations in tumor suppressor genes. However, tumor cells often need additional factors in the TME to maintain their survival, growth and angiogenic functions [95]. The TME is an important component of the invasive and metastatic potential of CRC cells [96]. The tumor matrix in the TME includes the extracellular matrix and a variety of host cells, including immune cells, vascular cells and mesenchymal cells [97]. Stromal cells, inflammatory cells and cancer cells communicate directly through cell contact and indirectly through paracrine signaling [98]. These signals include chemokines, with CCL5 involved in many mechanisms of cancer progression, including cell proliferation, migration, invasion, angiogenesis, metastasis and colonization and the regulation of the extracellular matrix and immune escape mechanism of cancer, as signaling [99,100]. The expression level of CCL5 is related to the growth and metastasis of many kinds of cancers [101–105], including CRC, and CCL5 not only plays an important role in the progression of CRC but can also be used to evaluate the curative effect of treatment and for diagnostics [106,107]. It may even predict severe hand and foot skin reactions in mCRC patients treated with repagenil [108].

In addition to acting directly on CRC cells as a tumor-promoting factor, CCL5 appears to be a regulator of inflammatory cell infiltration into tumor tissue [100]. Compared with that in control mice, the infiltration of CD8^+^ T cells into primary CRC tissues in CCL5^−/−^ mice was significantly increased. CCL5 deficiency upregulated the expression of PD-1 and PD-L1, and reduced drug resistance to anti-PD-1 antibody therapy. Knocking out CCL5 can lead to metabolic disorders in TAMs in the TME of CRC patients, which promotes the migration of CD8^+^ T cells in the TME [6]. TAMs are important to the TME and play roles in the occurrence and development of tumors by promoting the immune escape of tumor cells. CCL5 secreted by TAMs inhibits T cells and promotes tumor cell immune escape by stabilizing PD-L1 *in vitro* and *in vivo*. Mechanistically, CCL5 leads to the formation of the nuclear factor κρρa p65/STAT3 complex, which binds to the COP9 signal 5 (CSN5) promoter to upregulates CSN5 gene expression. Then, CSN5 regulates the deubiquitination and stability of PD-L1. High expression of CSN5 in CRC patients is associated with significantly shorter survival time [57]. The role played human mesenchymal stem cells in promoting CRC progression in the TME is due to the activation of epithelial-mesenchymal transition (EMT) mediated by the CCL5/CCR1/β-catenin/Slug pathway, indicating that CCL5 is an important factor in the regulation of CRC development through its mediation of stromal cell and cancer cell interaction [61]. In addition, chemokines secreted by mesenchymal cells may be important factors in the interaction between the TME mesenchymal cells and cancer cells. Specifically, the CCL3/4/5-CCR5 axis promotes tumor progression through the interactions between mesenchymal cells and CRC cells [97]. Jung-Yu Kan et al. [109] found that tumor-associated dendritic cells regulated the EMT and promoted the progression of CRC through CCL5-mediated Snail (a basic helix-loop-helix transcription factor) upregulation and downregulation in the protein expression of E-cadherin and other connective and adhesion proteins [109]. Moreover, CCL5 from tumor buds recruitment fibroblasts by acting on CCR5 receptors on fibroblasts. CCL5 derived from tumor buds also positively regulated the expression of solute carrier family 25 member 24 (SLC25A24) in fibroblasts, which may have activated the p Akt/pmTOR signal transduction pathway. In addition, CCL5 increased the number of fibroblasts in the TME, promoting tumor angiogenesis by enhancing VEGFA expression and fibroblasts transdifferentiation into vascular endothelial cells. Finally, the results showed that CCL5 promoted the synthesis of collagen in fibroblasts, promoting the progress of CRC [110]. Interestingly, the expression of CCL5 not only promoted the migration of Treg cells to tumors but also enhanced the ability of Treg cells to kill CD8^+^T cells [50]. In addition, CCL5 is an important factor in CRC cell immune escape by increasing the accumulation of bone marrow-derived suppressor cells during development CRC [111].

Increased expression of CCL5 has been found in the tumor tissues of CRC patients who drank alcohol. Further studies found that CCL5 enhanced autophagy in tumor cells and increased the migration ability of CRC cells by activating the AMPK signaling pathway, promoting the progression of CRC [112]. In addition, the expression of CCL5 in tumor tissues of patients with CRC may promote tumor invasion and lymph node metastasis [113]. *In vitro*, it was found that CCL5 promoted the migration of CRC cells and the proliferation of tumor cells in a dose-dependent manner [100]. In a mouse experiment, abnormal intestinal flora in NLRP6 inflammatory factor deficient mice induced colonic inflammation by inducing an increase in the level of chemokine CCL5, leading to the eventually development of CRC [114].

## 5. Promising clinical results indicate the use of CCL5 in colorectal cancer immunotherapy

CCL5 itself or the cells it recruits are closely related to the progression of CRC. Some studies have used CCL5 as a treatment index to evaluate CRC patients or cells recruited by CCL5 as carriers for targeted treatment in CRC patients. For example, CCL5 is involved in angiogenesis and is associated with the increased expression of vascular endothelial growth factor in cancer cells and vascular endothelial cells [115]. Therefore, in the NCT04397601 study performed by Ottaiano et al. CCL5 was regarded as an indicator to evaluate the efficacy of bevacizumab and aflibercept in the treatment of mCRC. In addition, an ongoing NCT04713891 study to evaluate the safety and antitumor activity of oral KF-0210 as a single drug in advanced solid tumor participants was also based on CCL5 as an evaluation indicator. In addition, genetic variations in CCL5 and CCR5 single nucleotide polymorphisms may predict the efficacy of first-line chemotherapy based on cetuximab in mCRC patients, as well as the efficacy of bevacizumab in mCRC patients [26,99]. Further study on how the CCL5/CCR5 signaling axis acts on host immune cells and cancer cells to create an antitumor and anticancer microenvironment may provide useful insights into options for future drug development [92]. Gene therapy has been widely studied in recent years, especially for the treatment of metastatic diseases. Mesenchymal cells in the TME are excellent gene carriers, in part because they are relatively easy to culture and expand *in vitro*. Importantly, these cells also show a significant natural tendency to migrate towards solid tumors [116]. Kerstin Knoop et al. used the sodium iodide transporter-encoding gene in mesenchymal stem cell-mediated tumor matrix-targeted radioiodine therapy for metastatic colon cancer [117]. Similarly, TAMs in the TME are excellent gene delivery vectors, and TAMs constitute the main cell type secreting CCL5. Considering the emerging TAM-targeting nanomaterials in recent years [118], TAM-targeting nanomaterials loaded with CCL5-neutralizing antibodies may be a promising strategy to inhibit the activity of TAMs/CCL5.

## 6. Conclusion

Generally, recent research results on the role played by CCL5 in the immune regulatory network in patients with CRC have been controversial. Some studies suggested that the secretion of CCL5 directly affects the proliferation, migration and survival of CRC cancer cells or indirectly recruit inflammatory cells into the TME, shaping the TME of CRC patients and playing a variety of roles in their own survival. In contrast, other studies have shown that the expression of CCL5 in CRC tissue exerts an antitumor effect, and its mechanism of antitumor immunity involves recruiting immune cells, mainly cDC1s and CD8^+^ T cells, to the TME. We believe that the main reason is because CCL5 binds many receptors, including CCR1, CCR3, CCR4 and CCR5.In addition, the recruitment rates of CCL5 secreted by different cells and the underlying mechanisms of action differ. After binding to a specific receptor, CCL5 triggers signaling through different downstream cascades. Although research on the role of CCL5 in the immune regulatory network in CRC patients has led to significant increases in understanding, determining the effects of changes to CCL5 actions in CRC patients, will provide further theoretical and practical directions for targeted immunotherapy strategies.

In the future, relevant studies on whether CCL5 is involved in the occurrence and development of colorectal cancer and tumor immunosuppression can be initiated with cells known to secrete CCL5 and by determining other kind of cells that secrete CCL5 and can be continued by studying the activation of downstream signaling pathways after the CCL5 interacts with CCR5. The relationships among pathway activation and tumor microenvironment changes, tumor angiogenesis, tumor cell invasion and metastasis, lymph node metastasis, immune cell recruitment and immunosuppressive cell recruitment are related to targeted pathways and related cell activation, providing ideas for clinical immunotherapy.

## Data Availability

The authors confirm that the data supporting the findings of this study are available within the article.
